# The hare syphilis agent is related to, but distinct from, the treponeme causing rabbit syphilis

**DOI:** 10.1371/journal.pone.0307196

**Published:** 2024-08-12

**Authors:** Petra Pospíšilová, Darina Čejková, Pavla Buršíková, Pavla Fedrová, Lenka Mikalová, David Najt, Nikola Tom, Linda Hisgen, Simone Lueert, Johannes T. Lumeij, Erik O. Ågren, Sascha Knauf, David Šmajs

**Affiliations:** 1 Department of Biology, Faculty of Medicine, Masaryk University, Brno, Czech Republic; 2 Department of Microbiology and Antimicrobial Resistance, Veterinary Research Institute, Brno, Czech Republic; 3 Department of Biomedical Engineering, Brno University of Technology, Brno, Czech Republic; 4 Institute of International Animal Health/One Health, Friedrich-Loeffler-Institut, Federal Research Institute for Animal Health, Greifswald, Insel Riems, Germany; 5 Division of Zoological Medicine, Department of Clinical Sciences of Companion Animals, Faculty of Veterinary Medicine, Utrecht University, Utrecht, The Netherlands; 6 Department of Pathology and Wildlife Diseases, National Veterinary Institute, Uppsala, Sweden; 7 Professorship for International Animal Health/One Health, Faculty of Veterinary Medicine, Justus Liebig University, Giessen, Germany; Suez University Faculty of Fish Resources, EGYPT

## Abstract

The treponemes infecting lagomorphs include *Treponema paraluisleporidarum* ecovar Cuniculus (*TP*eC) and ecovar Lepus (*TP*eL), infecting rabbits and hares, respectively. In this study, we described the first complete genome sequence of *TP*eL, isolate V3603-13, from an infected mountain hare (*Lepus timidus*) in Sweden. In addition, we determined 99.0% of the genome sequence of isolate V246-08 (also from an infected mountain hare, Sweden) and 31.7% of the genome sequence of isolate Z27 A77/78 (from a European hare, *Lepus europeaus*, The Netherlands). The *TP*eL V3603-13 genome had considerable gene synteny with the *TP*eC Cuniculi A genome and with the human pathogen *T*. *pallidum*, which causes syphilis (ssp. *pallidum*, *TPA*), yaws (ssp. *pertenue*, *TPE*) and endemic syphilis (ssp. *endemicum*, *TEN*). Compared to the *TP*eC Cuniculi A genome, *TP*eL V3603-13 contained four insertions and 11 deletions longer than three nucleotides (ranging between 6 and2,932 nts). In addition, there were 25 additional indels, from one to three nucleotides long, altogether spanning 36 nts. The number of single nucleotide variants (SNVs) between *TP*eC Cuniculi A and *TP*eL V3603-13 were represented by 309 nucleotide differences. Major proteome coding differences between *TP*eL and *TP*eC were found in the *tpr* gene family, and (predicted) genes coding for outer membrane proteins, suggesting that these components are essential for host adaptation in lagomorph syphilis. The phylogeny revealed that the *TP*eL sample from the European brown hare was more distantly related to *TP*eC Cuniculi A than V3603-13 and V246-08.

## Introduction

In addition to human pathogens such as *Treponema pallidum* ssp. *pallidum* (*TPA*), ssp. *pertenue* (*TPE*), and ssp. *endemicum* (*TEN*), causing syphilis, yaws, and bejel, respectively–the *Treponema* genus also contains other animal pathogens [1, 2]. Lagomorph syphilis is caused by *Treponema paraluisleporidarum* [3], with its ecovar Cuniculus (*TP*eC) and ecovar Lepus (*TP*eL), infecting rabbits and hares, respectively. Current knowledge centers almost exclusively around the *TP*eC strain Cuniculi A, isolated in 1939 in Maryland, USA [4], which is still maintained in the laboratory. *TP*eC strain Cuniculi A is genetically very related to the human pathogen *T*. *pallidum* (98% genome identity on the DNA level; [2, 5]). This study uses recently introduced name *Treponema paraluisleporidarum* [3], as this is consistent with the literature use in the last decade. However, since the GenBank database uses only validated names, the sequences were submitted as *Treponema paraluiscuniculi*. (*T*. *paraluiscuniculi* was validated decades ago and would not be eligible for validation according to current rules since it is highly related to *Treponema pallidum* [6]. Like human syphilis, *TP*eC causes sexually transmitted infections and known to be associated with skin ulcers in the anogenital region, the face, and/or the paws of rabbits (*Oryctolagus cuniculus* f. *domestica*) [7]. Sexually transmitted *TP*eL infections in European brown hares (*Lepus europaeus*) and mountain hares (*Lepus timidus*), are in most cases clinically inapparent or associated with orofacial and anogenital proliferative crusty skin lesions occurring at mucocutaneous junctions [7–9].

Based on serological screening studies that analyzed samples from more than 1500 animals, the average seroprevalence of syphilis in wild European lagomorphs ranges from 28% to 66%, depending on the serological test(s) used and the sample’s country of origin [10–14]. The prevalence of hare syphilis has no clear geographical gradient, however, it is known that seroprevalence against *TP*e*L* negatively correlates with altitude of sampling areas [11].

Genetic studies on the causative agent of lagomorph syphilis are limited and almost exclusively based on a single available genome sequence of the *TP*eC strain Cuniculi A, which is composed of circular chromosome of 1,133,390 bp with no plasmids [6]. So far, a single study [3] tested the genetic relatedness between *TP*eL and *TP*eC. The study revealed four nucleotide differences in the 2002 bp-long sequence obtained from partial sequences of the 16S rRNA gene, the DNA region downstream of the 16S rRNA gene, and the sequence within the *TPCCA_0225* gene including one nt difference found within the 16S rRNA genes [3].

Based on infection experiments conducted by Lumeij et al. [3] and on the genetic relatedness between *TP*eC and *TPA* [5, 6], we predicted that hare-infecting strains would be phylogenetically ancestral to rabbit-infecting strains and would be highly related to *TP*eC. In this study, we determined the complete genome sequence of *Treponema paraluisleporidarum* ecovar Lepus, isolate V3603-13. We used the previously described Pooled Sequence Genome Sequencing (PSGS) technique to compare the V3603-13 genome to the once-determined genomic sequence *TP*eC strain Cuniculi A. We found that the treponeme causing hare syphilis was in many ways similar but distinct from the treponeme causing rabbit syphilis.

## Materials and methods

### Lagomorph samples

We obtained samples from mountain hares (*Lepus timidus*) hunted in Sweden. *TP*eL sample V3603-13 was obtained from a hare sampled close to Enånger in county Gävleborg (latitude 61.32470, longitude 17.02430) in 2013. Sample V246-08 came from a hare sample hunted in 2008 near Orsa in the county of Dalarna (latitude 61.30180, longitude 14.28340). Both samples were taken from hares submitted within the Swedish general (passive) wildlife disease surveillance program, where hunters or anybody else can report findings of disease or mortality in wildlife. The hares were shot by hunters and when they noted scabs and lesions on the lips and nose, they contacted Swedish Veterinary Agency (SVA). Upon request from SVA, the carcasses were sent to the institute for diagnostic examinations. No further information is available on the location of lesions. The brown hare isolate, Z27 A77/78, was collected in The Netherlands in 2010 and was described in an earlier study [3].

### Sampling and extraction of DNA

Samples from the skin lesions (full skin thickness tissue samples) were taken from dead animals and stored in a -80 degree Celsius biobank freezer until thawed and used in this study. DNA was extracted from tissue material using the QIAamp DNA Mini Kit (Qiagen, Hilden, Germany) following the manufacturer’s protocol with some minor modifications. Briefly, we extracted DNA from tissue samples according to the protocols published by Hisgen et al. [11]. Subsequently, glycogen precipitation was performed to clean and concentrate the DNA as described by Knauf et al. [15]. We measured the DNA yield using a NanoDrop photometer (Thermo Fisher Scientific, Darmstadt, Germany).

### Amplification of genomic DNA

The genomic DNA of all three samples was amplified using the multiple displacement amplification approach (REPLI-g kit, QIAGEN, Valencia, CA, USA). Samples were diluted one to 50 times (depending on the positivity of treponemal-specific PCR amplification from undiluted and diluted samples) and then used as a template for PSGS, as previously described [16–18]. The DNA was amplified using PrimeSTAR GXL DNA Polymerase (Takara Bio Inc., Otsu, Japan) with 133 specific primer pairs (published by Mikalová et al. [19], with minor modifications) to obtain overlapping PCR products covering the entire chromosome. PCR products were amplified using touchdown PCR under the following cycling conditions: initial denaturation at 94°C for 1 min; 8 cycles: 98°C for 10 s, 68°C for 15 s (the annealing temperature was gradually reduced at a rate of 1°C per cycle), and 68°C for 6 min; 35 cycles: 98°C for 10 s, 61°C for 15 s, and 68°C for 6 min (43 cycles in total); followed by the final extension at 68°C for 7 min. PCR products were subsequently purified using QIAquick PCR Purification Kits (QIAGEN, Valencia, CA, USA) and mixed in equimolar amounts to produce four distinct pools, which were then used for whole genome sequencing.

### Whole genome sequencing and assembly of the genomes

The sequencing library for *TP*eL strain V3603-13 was prepared from 1 ng of RNA-free genomic DNA using a Nextera XT DNA Sample Preparation kit (Illumina, CA, USA). Sequencing was performed using the MiSeq Reagent Kit v3 in the MiSeq system (Illumina, USA) at the Veterinary Research Institute sequencing facility (Brno, Czech Republic). The sequencing reads of individual pools were handled separately and assembled *de novo* with SeqMan NGen v4.1.0 software (DNASTAR, Madison, WI, USA) using the default parameters. Contigs obtained from *TP*eL strains were then aligned to the genome of the *TP*eC strain Cuniculi A (CP002103.1) using Lasergene software (DNASTAR, Madison, WI, USA). Missing parts of genomes were subsequently Sanger sequenced (GATC Biotech, Germany). In addition, MinION sequencing (Oxford Nanopore Technologies (ONT), Oxford, UK) was used to sequence paralogous gene regions, mainly those containing *tpr* genes.

The number of repetitions within the *arp* gene (*TP0433*) was determined by amplification of PCR products with primers 32BrepF1 (5′-CGT TTG GTT TCC CCT TTG TC-3′) and 32BrepR1 (5′-GTG GGA TGG CTG CTT CGT ATG-3′) as described by Harper et al. (2008); the resulting PCR products were subsequently Sanger sequenced. Repetitive sequences within the *TP0470* gene were amplified and sequenced using primers TPI34F4 (5′-GTC TTG TGC ACA TTA TTC AAG-3′) and TPI34R5 (5′-CTT CGT GCA ACA TCG CTA CG-3′).

Both the V246-08 and Z27 A77/78 genomes were amplified using the PSGS method and then sequenced using the Illumina platform as described for the V3603-13 sample. The quality of the raw reads was checked using FastQC [20]. Raw reads were trimmed in length (a minimal length of 35 nt) and quality (Phred quality score ≥ 20) using Cutadapt [21]. Preprocessed reads were mapped using the BWA MEM algorithm [22] onto the *TP*eL Cuniculi A genome (GenBank Acc. No. CP002103.1). The Cuniculi A reference genome was divided into four parts representing the "pools" described above. The post-processing of the reads’ mapping was performed using Samtools [22], Picard (https://broadinstitute.github.io/picard/), GATK [23], and NGSUtils/bamutils [24]. Low-quality mappings were omitted from analyses (i.e., mapping quality; MAPQ < 40) as well as reads mapping to multiple sites (e.g., in repetitive and paralogous regions). A minimal alignment length was set to 35 bps, the maximum number of allowed mismatches was set to 5 (or 5% of the read length), and the maximum soft-clipping was set to 5% of the read length. Consensus sequences were determined using variants detected using Samtools "variant calling" followed by Vcfutils [22] and Seqtk (https://github.com/lh3/seqtk). The breadth and depth of coverages were calculated using GATK software.

Libraries used for MinION (ONT) sequencing were prepared using the 1D Native barcoding genomic DNA protocol with EXP-NBD103 and SQK-LSK108 kits (ONT) and Nanopore MinION Spoton flow cells (FLO-MIN106D, version R9) and sequenced for 48 hours. Base-calling and barcoding were done using Guppy (v4.4.1, ONT) in high-accuracy base-calling mode and q-score 7.

### Construction of phylogenetic tree

To determine evolutionary relationships among pathogenic treponemes, corresponding parts of the available treponemal genomes (orthologous sequences) were used and included *TP*eC Cuniculus strain Cuniculi A (CP002103.1), *TP*eC Cz-2020 (MW323408), and *TP*A strain SS14 (CP004011.1). The phylogenetic tree was constructed using the Maximum Likelihood method based on the Tamura-Nei model and MEGA 7 software, which was also used for model testing [25]. Bootstrap values were equal to 1000.

### Nucleotide sequence accession number

The complete genome sequence of the *TP*eL V3603-13 isolate was deposited in GenBank under accession number CP097901, Bioproject PRJNA606433. Since *Treponema paraluisleporidarum* ecovar Lepus is not validated, the sequences were submitted as *Treponema paraluiscuniculi* into the GenBank database. This is the reason why sequence CP097901 was submitted as *Treponema paraluiscuniculi* strain L2, which was the original designation of the isolate. The sequencing reads assembled into draft genome sequences of the *TP*eL V246-08 (JAMZQX000000000) and Z27 A77/78 isolates were deposited in GenBank under Bioproject accession numbers PRJNA837879 and PRJNA837886, respectively.

## Results

### Sequencing of *TP*eL isolates

*TP*eL isolates were sequenced using PSGS, as described earlier [16, 17]. Briefly, DNA was amplified with 133 pairs of specific primers to obtain overlapping PCR products [18]). To facilitate sequencing of paralogous genes containing repetitive sequences, PCR products were mixed in equimolar amounts into four distinct pools. The PCR products constituting each pool were labeled with multiplex identifier (MID) adapters and Illumina sequenced. Pools 1 through 4 corresponded to PCR amplicons grouped largely by their location in four genome segments. Sequencing results of *TP*eL isolates, including V3603-13, V246-08, and Z27 A77/78 samples, are shown in Table 1. Sequencing of *TP*eL V3603-13 resulted in 1,247,184, 1,481,259, 979,180, and 1,224,430 mapped deduplicated reads for pools 1, 2, 3, and 4, respectively. The *TP*eL V3603-13 genome was completely determined, leaving no sequencing gaps or ambiguities. Sequencing of *TP*eL V246-08 revealed 210,955, 624,319, 603,376, and 1,062,714, and Z27 A77/78 revealed 529,826, 769,416, 762,979, and 380,436 of mapped reads without duplicates in pools 1, 2, 3, and 4, respectively. Compared to the genome of *TP*eL strain V3603-13, 99.0% and 31.7% of the total genome of V246-08 and Z27 A77/78, respectively, were determined.

**Table 1:** Sequencing of *TP*eL isolates and the corresponding sequencing parameters.

	Breadth/depth of coverage (no. determined kb/average coverage)				
Isolate	Pool 1	Pool 2	Pool 3	Pool 4	Genome
**V3603-13**	253.56/	243.93/	251.98/	379.62/	1129.05/
	1044	1414	905	762	998
**V246-08**	249.63/	246.26/	251.79/	373.99/	1,121.67/
	201	1249	1206	1208	992
**Z27 A77/78**	52.53/	105.03/	90.38/	111.37/	359.3/
	615	913	905	608	773

### The overall genome structure of *TP*eL isolate V3603-13

The complete genome sequence of *TP*eL isolate V3603-13 comprised 1,132,489 nucleotides with no sequencing gaps or ambiguities. The genome sequence also includes variable sites in the *tprK* gene (*TP0897*), where the most common variant (with a frequency greater than 50%) was used for the final whole genome consensus sequence. The *TP*eL V3603-13 genome is 901 nt smaller than the reference genome of *TP*eC strain Cuniculi A (GenBank acc. number CP002103). There is overall gene synteny with the *TP*eC strain Cuniculi A genome and the human pathogen *T*. *pallidum* (subsp. *TPA*, *TPE*, and *TEN*). The *TP*eL V3603-13 genome showed a different *rrn* spacer pattern compared to the *TP*eC Cuniculi A genome, i.e., tRNA gene for Ala/Ile in V3603-13 compared to tRNA gene for Ile/Ala in the rabbit infecting strain of Cuniculi A. Sequences of the 5S, 16S, and 23S rRNA genes were identical in both operons and were without macrolide resistance coding single nucleotide changes A2058G or A2059G in the 23S rRNA genes [26, 27]. The number of 60 bp-long repetitions within the *arp* gene (*TP0433*) in *TP*eL V3603-13 and *TP*eC strain Cuniculi A genomes differed and was 19 and 21 repetitions, respectively. The number of 24 bp-long repetitions was 19 in the *TP0470* in *TP*eL V3603-13, and 6 in *TP*eC strain Cuniculi A. Table 2 summarizes the overall genome parameters of *TP*eL isolate V3603-13.

**Table 2:** Genomic features of the *TP*eLV3603-13 isolate compared to *TP*eC strain Cuniculi A.

Genome parameter	*TP*eL V3603-13	*TP*eC Cuniculi A
Genome size	1,132,489 bp	1,133,390 bp
G+C content	52.7%	52.8%
No. of predicted genes	1067, including 54 untranslated genes	1070, including 54 untranslated genes
No. of genes encoded on plus/minus DNA strand	577/490	577/493
Average/median gene length	1007/873 bp	1006/873 bp
Intergenic region length	60,749 bp (5.4% of the genome length)	62,494 bp (5.5% of the genome length)
No. of predicted genes encoding proteins similar to proteins of known function	647	650
No. of genes encoding conserved hypothetical proteins	137	139
No. of genes encoding hypothetical proteins	229	227
No. of pseudogenes or gene fragments	55	51
No. of tRNA loci	45	45
No. of rRNA operons	2* (6 genes)	2* (6 genes)
No. of other stable RNAs	3	3

*TPeL V3603-13 and *TP*eC Cuniculi A genomes showed different *rrn* spacer patterns (Ala/Ile and Ile/Ala).

### Major sequence differences between *TP*eL V3603-13 and *TP*eC Cuniculi A

**Fig 1:**
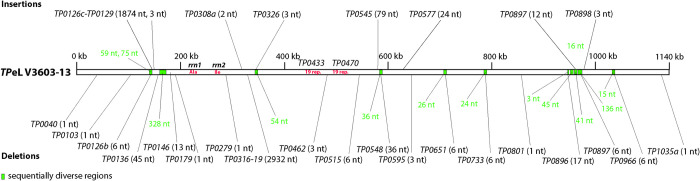
An overview of genome differences between *TP*eL V3603-13 and *TP*eC Cuniculi A. Insertions and deletions are shown above and below the schematic representation of the *TP*eL isolate V3603-13 chromosome, respectively. Green areas represent sequentially diverse regions, and the lengths of these regions are shown in green letters, while indel lengths are shown in black letters.

Despite the overall genome synteny of *TP*eL V3603-13 and *TP*eC strain Cuniculi A, several genetic differences differentiate the genomes, including indels and single nucleotide variants (SNVs). An overview of the genetic differences between the rabbit infecting strain, Cuniculi A, and the V3603-13 genome is shown in Fig 1 and S1 Table. While most of the indels were found within genes, a minority was found in the intergenic regions (IGR). Moreover, most of the indels in the genes did not change the reading frame leading to indels of one to few amino acids. Compared to the *TP*eC strain Cuniculi A genome, *TP*eL V3603-13 contains four insertions greater than three nucleotides: a 1,874 nt-long insertion containing a *TP0126c*-*TP0129* fragment similar to the orthologous *TPA* strain Philadelphia 1 genomic sequence [28], a 79 nt insertion in the IGR between *TP054*5-*TP0546* similar to the *TPA* strain Philadelphia 1 genomic sequence, a 24 nt insertion in *TP0577* that is similar to the *TEN* strain 11q/j genomic sequence [29], and a 12 nt insertion (with undetected sequence similarity) in *TP0897* (*tprK*) (S1 Table).

Compared to the *TP*eC strain Cuniculi A genome, the *TP*eL V3603-13 genome contained eleven deletions greater than three nucleotides: a 2,932 nt deletion similar (but larger) to the deletion present in *TEN* strains [29] comprising the *tprFG* and *tmpC* (*TP0319*) genes, a six nt-long deletion in *TP0126b*, a 45 nt deletion in *TP0136*, a 13 nt deletion in *TP0146*, a six nt deletion in *TP0515*, a 36 nt deletion in *TP0548*, a six nt deletion in the IGR between *TP0650*–*TP0652*, a six nt deletion in *TP0733*, a 17 nt deletion in the IGR between *TP0895*–*TP0897*, a six nt deletion in *TP0897* (*tpr*K), and a six nt deletion in *TP0966* (S1 Table).

In addition, there were 25 additional indels with lengths of 1–3 nucleotides, altogether spanning 36 nt (S1 Table). Three of these changes lead to in-frame insertions or deletions. Other indels located in genes lead to frameshifts, which are shown in S1 Table. A subset of insertion/deletion differences described in S1 Table was also found in *TP*e*L* isolate V246-08 supporting lack of sequencing artifacts.

Several sequentially diverse chromosomal regions (defined as multiple SNVs) were identified in *TP*eL V3603-13 genome: a 59 nt long region in *TP0126b* showing similarity to *tprK* of *TPA* SS14, a 75 nt long region in *TP0126b* showing similarity to *tprK* of *TPA* X-4, a 328 nt long region in *TP0136* with similarity to *TP0134* of *TPA* Philadelphia 1, a 54 nt long region in position of *TP0326* similar to *bamA* of *TPA* CZ_177zB (*TPA* Nichols-like strain) [30], a 36 nt long region not similar to any known treponemal sequences in position of *TP0548*, a 26 nt long region similar to IGR *TP0650*-*TP0652* of *TPA* Philadelphia 1 in position of IGR *TP0650-652*, a 24 nt long region not similar to any known treponemal sequences in position of *TP*0733, four regions upstream and in the *tprK* gene (3nt, 45 nt, 41nt, 136 nt) similar to *TP*eC or *TPA* SS14 sequences, and a 15 nt long region similar to *TPE* Fribourg-Blanc in position of *TP0966* (S1 Table).

Moreover, frameshift mutations resulting in two new pseudogenes (*TP0308a*, *TP1035a*) and major sequence changes, including protein elongation or shortening (*TP0040*, *TP0179*, *TP0279*, *TP0471*, *TP0778*, *TP0801*) were described. In addition, another frameshift mutation in existing pseudogenes was found in two pseudogenes (*TP0103*, *TP0146*) (S1 Table). Lastly, the *mglB* gene (*TP0545*) of *TP*eL V3603-13 appears to be fully functional and similar to *TPE* strains (due to the insertion of 79 nts in IGR between *TP0545–TP0546*). Similarly, a nucleotide insertion in V3603-13 *TP0617a* results in a gene version identical to the paralogous *TP0315* gene in the V3603-13 genome. Genes *TP0651* and *TP0896* in V3603-13 are longer but similar to the versions present in *TPE* strains.

**Fig 2:**
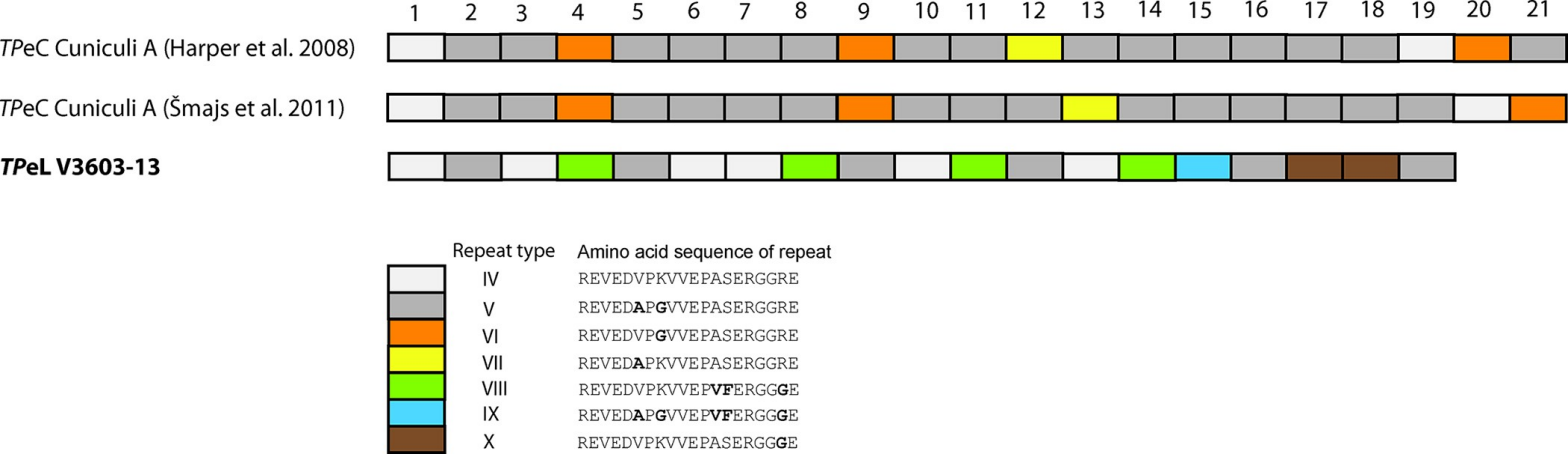
Composition of repeat motif regions observed in the *TP*eL V3603-13 and *TP*eC Cuniculi A genomes. Note that repeat motifs determined by Harper et al. [31], and by Šmajs et al. [6], differ, likely due to repeat reshuffling during individual laboratory handling of the Cuniculi A strain.

As shown in Fig 2, repeat motifs within the *arp* gene (*TP0433*) and the arrangement of the repeat sequences differ between *TP*eL V3603-13 and *TP*eC Cuniculi A genomes.

### Single nucleotide sequence differences between the *TP*eL V3603-13 and *TP*eC strain Cuniculi A genomes

In addition to the described differences, 309 additional nucleotide variants are dispersed throughout the genome in *TP*eL V3603-13 compared to the *TP*eC Cuniculi A reference genome. These nucleotide differences were primarily represented by single nucleotide variants (SNVs) and less frequently by double nucleotide differences (n = 8, covering 16 nucleotides). Analysis of the SNVs between *TP*e*L* samples V3603-13 and V246-08 revealed altogether 35 single nucleotide variants indicating that majority of genetic differences between hare and rabbit syphilis is shared by both V3603-13 and V246-08 samples.

### The phylogenetic relationship of lagomorph and human pathogenic *Treponema*

**Fig 3:**
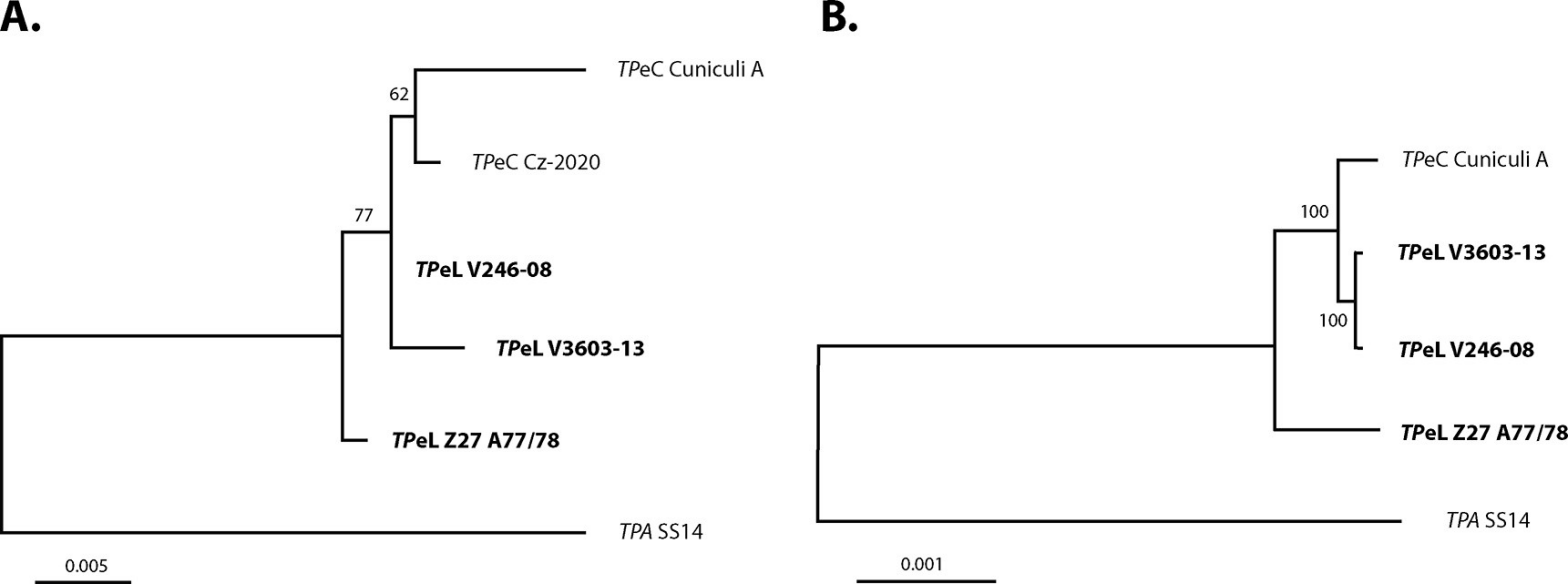
Molecular phylogeny of *TP*eL and *TP*eC samples. **A.** The tree was built on partial *TP0548* sequences (*TP*eC Cz-2020 and *TPA* SS14) [32, 33]. There was a total of 777 positions in the final dataset. **B.** The tree was built on available genome sequences. There were 355,133 positions in the final dataset. The trees were constructed using the Maximum Likelihood method based on the Tamura-Nei model. Bootstrap support is shown next to the branches. The scale shows the number of substitutions per site. As an outgroup, the *TPA* SS14 sequence was used. All positions containing gaps and missing data were omitted.

To determine the genetic relatedness of *TP*eL/*TP*eC samples, a tree was built on partial *TP0548* sequences available for two *TP*eC strains/isolates (Cuniculi A; Cz-2020) and three *TP*eL isolates *TP*eL V3603-13, V246-08, and Z27 A77/78 (Fig 3A). Sample Cz-2020 is the only other known member of *TP*e*C* and only sequences of few loci are available. Therefore, locus TP0548 was selected as the only locus with available sequences in all 5 *T*. *paraluisleporidarum* samples. *TP*eL isolates clustered together but separate from *TP*eC isolates. To determine the genetic relatedness of both ecovars using a more robust analysis, a tree was built on partial genome sequences available for the complete genome of *TP*eL V3603-13 and *TP*eL isolates V246-08 and *TP*eL Z27 A77/78. Altogether, about one-third of the genome length (355,133 nucleotides) was used to construct the phylogenetic tree (Fig 3B). Both *TP*eL isolates V3603-13 and V246-08, which were isolated from *L*. *timidus*, clustered closely with the *TP*eC Cuniculi A genome. At the same time, the Z27 A77/78 sample, taken from the *L*. *europeus* in The Netherlands [3], was more distantly related.

## Discussion

In this study, we have determined the first whole genome sequence of the causative agent of hare syphilis. *TP*eL isolate V3603-13 was obtained from an infected Swedish mountain hare. In addition, we generated two partial genome sequences for *TP*eL isolate V246-08, also from an infected mountain hare in Sweden, and Z27 A77/78 that came from a European brown hare in The Netherlands [3]. While the sample quality of V246-08 and Z27 A77/78 (quality refers to the amount of available treponemal DNA) did not allow us to sequence the whole genome, considerable parts of the corresponding genomes were sequenced with the shortest determined sequence from Z27 A77/78 being equal to about a third of the *TP*eC strain Cuniculi A genome, i.e., 31.7%, 355,133 nucleotides.

Until now, only a limited number of studies have been published on the genetics of *TP*eL [3, 34] even though infections caused by *TP*eL appear to be widespread among European hares [11–14, 35]. Since most of the previous and current *TP*eL infections are subclinical and detectable only with serology and/or direct detection of *TP*eL DNA [8], the scarcity of clinical symptoms could explain the lack of scientific data on treponemal infections in hares.

The genome of *TP*eL V3603-13 comprised of 1,132,489 nt and is the smallest genome determined to date of the group of closely related pathogenic treponemes, including human pathogenic *TPA*, *TPE*, and *TEN*. At the whole genome level, genetic similarity between *TP*eL and *TP*eC was > 99.8%, with almost complete gene synteny between the genomes. Interestingly, both the *TP*eL V3603-13 and *TP*eC Cuniculi A genomes showed different *rrn* spacer patterns (Ala/Ile and Ile/Ala, respectively), a feature previously noticed within *TPA* and *TPE* strains as a result of reciprocal intragenomic translocation events [2, 34]. The number of repetitions within the *arp* gene (*TP0433*) in *TP*eL V3603-13 was less than *TP*eC Cuniculi A (19 vs. 21), and the gene was composed of different sequence motifs compared to the *arp* of Cuniculi A (Fig 2) [31]. Among *TPA* isolates, the number and the structure of the *arp* repeats were shown to generally correlate with whole genome phylogeny [36]. Interestingly, the Cuniculi A strain *arp* gene analyzed by Strouhal et al. [5] and Šmajs et al. [6] differed in the sequence of repeat motifs from the same strain analyzed by Harper et al. [31], suggesting that these gene repeat components are prone to frequent reshuffling and accumulation of point mutations. Consistent with this observation, previous studies on the number of *arp* repeat motifs among *TPA* isolates revealed differences in the repeat numbers between whole blood and swab samples taken from the same patient [37], again suggesting the genetic plasticity of this locus. Both the *TP*eC and *TP*eL *arp* genes contained multiple repeat motifs (Cuniculi A, n = 4; V3603-13, n = 5), a feature that has only been described for *TP*A and *TP*eC strains but not for *TPE* (including stains infecting nonhuman primates [38] and *TEN* strains [31].

One major difference between the rabbit infecting *TP*eC Cuniculi A and the hare *TP*eL V3603-13 genome was the presence of an 1874 nt-long insertion in V3603-13. This insertion mainly corresponded to the *TP0126c*-*TP0129* genes in other *TPA* and *TPE* treponemes, which suggests that the corresponding region was deleted during the evolution of the Cuniculi A strain. In a subpopulation of the *TPA* Nichols strain, a 1.3-kb deletion at this genomic locus (i.e., in *TP0126*) was found in an earlier study [39]; both the *TPA* Nichols and *TP*eC Cuniculi A deletions overlapped by 376 nt. Another previous study revealed that *TP0126*, *TP0126b*, *TP0126c*, *TP0127d*, *TP0128*, and *TP0130* (in addition to other genes) have a modular genetic structure enabling rapid genetic diversification of treponemal strains [40]. At the same time, these regions, due to the presence of repetitive sequences, may also have less genetic stability and be prone to deletion events. Since the Nichols strain has been propagated in rabbits for some time, the deletion of this region may represent one of the adaptations to infecting rabbits (or hares). At least two additional insertions (in *TP0545-546* and *TP0577*) in the V3603-13 genome matched sequences present in other *TPA* and *TPE* strains, suggesting additional deletions in the evolution of the rabbit pathogen, *TP*eC. Based on cross-infection experiments [3], it was predicted that *TP*eL can infect both rabbits and hares while *TP*eC infects only rabbits. Therefore, the deleted regions in *TP*eC may be important relative to hare infections. However, genome manipulation experiments would be needed to prove this and better understand the host-pathogen evolution of treponemal pathogens.

Another striking feature of the *TP*eL V3603-13 genome is a 2932 nt-long deletion in V3603-13 affecting *tprFG* and *tmpC* (*TP0319*) genes (compared to *TP*e*C* strain Cuniculi A). The genome of *TP*eC Cuniculi A has a similar but smaller deletion in this region (compared to *TPA* strain Nichols), suggesting the independent origin of these deletions. Moreover, the surrounding genes, including *TP0309-311*, *TP0313*, *TP0315*, and *TP0318*, contain frameshift mutations in both *TP*eC Cuniculi A and *TP*eL V3603-13 genomes, and it is therefore possible that the entire region is not needed for infection of lagomorphs, including both rabbits and hares. Yet, it is not clear if the *tprFG* region is required for infection of humans since similar but smaller deletions in the *TEN* strains were found in subpopulations of individual *TEN* strains that were experimentally propagated in rabbits [17, 41]. On the other hand, the *TEN* strains isolated directly from Cuban patients suspected of having syphilis [42] contained a deletion at the *tprFG* locus [43] which was similar to one described in the *TEN* reference strains Bosnia A and Iraq B [17, 41]. This suggests that the deletion does not preclude infection in humans nor the emergence of early syphilis-like symptoms [29, 42, 44].

**Fig 4:**
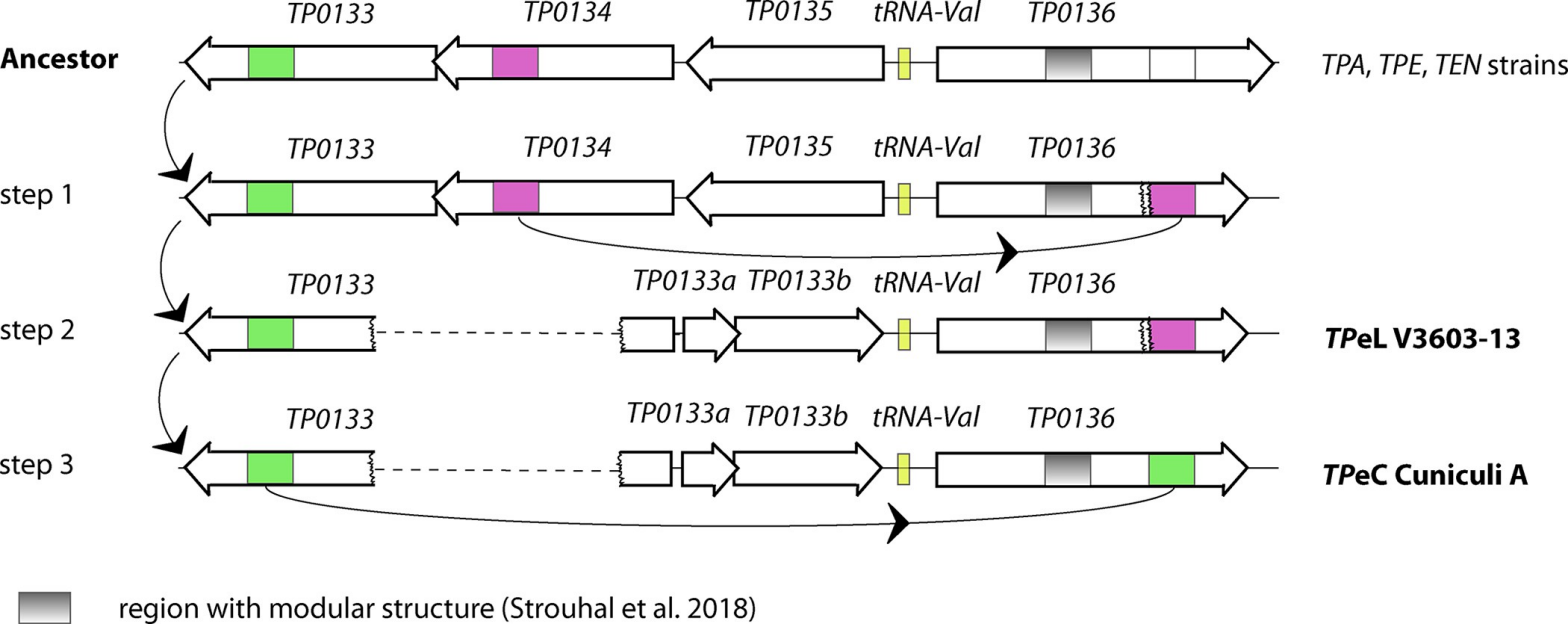
A schematic representation of a possible evolutionary model of the *TP0136* locus in *TP*eL and *TP*eC treponemes. The evolution of this region required several steps, including two gene conversion events and one deletion. The part of *TP0136* showing a modular structure was not affected during these changes.

Analysis of the *TP0136* gene in the V3603-13 genome revealed a 328 nt-long region similar to *TP0134* in *TPA* Philadelphia 1 [28] while the *TP0136* gene in Cuniculi A had a sequence similar to *TP0133* (Fig 4). Since the *TP0134* gene (including the donor sequence) was deleted from both the *TP*eC Cuniculi A and *TP*eL V3603-13 genomes, this finding cannot be explained by sequence gene transfer from the *TP0134* locus. An explanation is the presence of a common ancestor, i.e., for the *TP*eC Cuniculi A and *TP*eL V3603-13 strain, with a *TP0134*-like sequence at the *TP0136* locus (Fig 4). Subsequently, a deletion might have occurred. The lineage then became ancestral to the *TP*eL genome while additional gene conversion leading to replacement of the *TP0134*-like sequence by the *TP0133*-like sequence occurred during the evolution of the rabbit infecting *TP*eC genome of Cuniculi A (Fig 4). Although this model suggests *TP*eC being an evolutionarily modern version of *TP*eL, other alternative explanations, including gene recombination events [45] are also possible.

The *TP0136* locus encodes for the fibronectin-binding protein [46] and is known to be a recombinant and positively selected locus in *TPA* treponemes [28, 47]. Moreover, this locus has been predicted to have a modular structure that can lead to relatively frequent sequence changes and indels [40, 48]. However, the part of *TP0136* with the modular structure was not affected during the above-described replacements (Fig 4). All this evidence points to the importance of the *TP0136* locus during human and animal infections.

Besides deletions, insertions, and stretches of nucleotide sequences resulting from gene conversions, we found 309 single nucleotide variants (SNVs) between the *TP*eC Cuniculi A and *Tp*eL V3603-13 genomes. These differences were present in single or two nt sequences (n = 8), suggesting that these events represented accumulated mutations. Although mutation rates in *TP*eL and *TP*eC remain unknown, previous studies estimated the mutation rates of the genetically related treponemes *TPA* and *TPE*. The mutation rate was estimated to be 2.8–4.1 × 10^−10^ per site per generation [49, 50], which corresponds to a mutation rate of 0.846–1.21 × 10^−7^ per nucleotide site per year. Other studies, based on temporal analyses with relaxed clock models, estimated slightly higher treponemal mutation rates, i.e., 3.02 × 10^−7^ [51] and 6.6 × 10^−7^ [52] per nucleotide site per year, which would correspond to 0.5–3 kiloyears of separate evolution for lagomorph treponemes. We note here that these mutation rate estimates, based on *TPA*/*E* strains in humans, do not necessarily correspond to lagomorph infecting *TP*eC/L. Moreover, mutation rates could vary over time, especially during the early phases of adaptation to a new host (species), and mutation rates could be even lower in *TPA/TPE* [18, 50].

The mutations causing resistance to macrolide antibiotics (i.e., A2058G or A2059G mutations in the 23S rRNA genes [26, 27, 53]) were not found among the analyzed *TP*eL isolates. *TP*eL strains and *TP*eC strain Cuniculi A remain the only group of pathogenic treponemes where these mutations have not been detected, in contrast to *TPA* [26, 27], *TPE* [54, 55], and *TEN* [56, 57]. The absence of macrolide resistance in *TP*eL and *TP*eC is consistent with the absence of macrolide resistance in *TPE* isolated from wild nonhuman primates [15, 38, 58, 59]. Although speculative and theoretical resistance could quickly emerge in wildlife, too, particularly under selection pressure caused by the mass administration of antimicrobial treatments.

Functionally, the most noticeable differences were found in *tpr* genes (*tprF*, -*G*, -*K*), genes that encode for outer membrane proteins/antigens (*TP0136*, *tmpC*, *TP0326*, *TP0433*, *TP0470*, *TP0471*, *TP0515*, *TP0548*, *TP0651*, *TP0733*, and *TP0966*), and transporter/chemotaxis proteins (*TP0040*, *TP0146*, *TP0545*, and *TP0577*). Genes coding for metabolic functions (*TP0179*, *TP0279*, *mazG*, and *TP0801*) and hypothetical proteins (*TP0126b-129*, *TP0308a*, *TP0617a*, *TP0896*, and *TP1035a*) were relatively infrequent. Out of these functional groups, outer membrane proteins appear to be the most important part of the proteome, i.e., they facilitate immune evasion and adaptation to different hosts.

## Conclusions

We determined the first complete genome sequence of *Treponema paraluisleporidarum* ecovar Lepus (*TP*eL V3603-13) isolated from a naturally infected mountain hare in Sweden and provided draft genome sequences of two additional *TP*eL strains. The agent causing hare syphilis was found to be similar but distinct from the rabbit syphilis treponeme; the major predicted proteome differences were associated with the Tpr proteins and outer membrane proteins/antigens. Based on previous estimations of *TPA* and *TPE* mutation rates, both *TP*eL and *TP*eC appear to be separated by 0.5–3 kiloyears of lagomorph treponeme evolution. However, more samples from naturally infected hares and rabbits need to be analyzed to understand better the genetic diversity of lagomorph syphilis and the phylogeny of lagomorph treponemes.

## Supporting information

S1 Table Genetic differences found in the *TP*eL V3603-13 genome when compared to the *TP*eC Cuniculi A genome. (DOCX)
