# Supplementary material for: The hare syphilis agent is related to, but distinct from, the treponeme causing rabbit syphilis
Source: PLoS One. 2024 Aug 12;19(8):e0307196. doi: 10.1371/journal.pone.0307196 (PMC11318916; doi:10.1371/journal.pone.0307196)
Supplement: S1 Table — (DOCX) [file pone.0307196.s001.docx]

**S1 Table.** **Genetic differences found in the *TP*eL V3603-13 genome when compared to the *TP*eC Cuniculi A genome.** Only changes resulting in larger amino acid replacements (defined as two and more amino acid changes) are shown. The single nucleotide variants (SNVs) (n= 309) are not shown.

| **Gene** | **Gene name** | **Gene/protein function** | **Type of difference** | **Remark/reference** |
| --- | --- | --- | --- | --- |
| *TP*0040 |  | methyl-accepting chemotaxis protein | frameshift (deletion, 1 nt) | protein shortening of 6 amino acids (aa) at the C-terminus |
| *TP*0103 | ***recQ*** | A*TP*-dependent helicase RecQ | frameshift (deletion, 1 nt) | frameshifts in both V3603-13 and *TP*eC |
| *TP*0126b |  | hypothetical protein | deletion (6 nt)  MSC* (75 nt) | region similar to *tprK* of *TP*A X-4 |
| *TP*0126c-*TP*0129 |  | hypothetical proteins | insertion (1874 nt) | similar to *TP*A Philadelphia 1, this DNA region is not present in *TP*eC but is present in other treponemes |
| *TP*0136 |  | fibrinogen-binding outer membrane protein | deletion (45 nt)  MSC (328 nt)  MSC (36 nt) | Brinkman et al. 2008, 15 aa  similar to *TP*0134 of *TP*A Philadelphia 1 while *TP*eC is similar to the *TP*0133 sequence  total of 60 aa replacements |
| *TP*0146 |  | CHR family chromate ion transporter | deletion (13 nt) | frameshifts in both V3603-13 and *TP*eC |
| *TP*0179 |  | Fe-only hydrogenase** | frameshift (deletion, 1 nt) | protein shortening of 22 aa at the N-terminus |
| *TP*0279 |  | bifunctional cytidylate kinase/ribosomal protein S1 | frameshift (deletion, 1 nt) | protein elongation of 16 aa at the N-terminus |
| *TP*0308a |  | hypothetical protein | frameshift (insertion, 2 nt) | not present in *TP*eC |
| *TP*0316-319 | ***tprF,G, tmpC*** | *TP*r protein F,G; basic membrane protein | deletion (2932 nt) | similar to deletion in TEN Iraq B, but larger |
| *TP*0326 | ***tp92*** | outer membrane protein | MSC (54 nt) | region similar to *bamA* of *TP*A CZ_177zB |
| *TP*0433 | ***arp*** | acidic repeat protein | deletion (120 nt) | 19 repetitions (60-bp long) instead of 21 |
| *TP*0462 |  | conserved hypothetical protein | deletion (3 nt)  MSC (34 nt)  MSC (54 nt) | deletion of 1 aa  no similarity to other treponemal sequences, total of 22 aa replacements |
| *TP*0470 |  | tetratricopeptide repeat containing protein** | insertion (312 nt) | 19 repetitions (24-bp long) instead of 6 |
| *TP*0471 |  | tetratricopeptide repeat containing protein** | nucleotide change in start codon (1 nt) | protein shortening of 12 aa at the N-terminus |
| *TP*0515 |  | organic solvent tolerance protein | deletion (6 nt) | deletion of 2 aa |
| *TP*0545 | ***mglB*** | sugar ABC superfamily A*TP* binding cassette transporter, binding protein | IGR and gene insertion (79 nt) | sequence version similar to *TP*E strains, deletion of this region is present only in Cuniculi A  protein elongation of 11 aa at the N-terminus |
| *TP*0548 |  | FadL-like protein**** | deletion (36 nt)  MSC (22 nt) | deletion of 12 aa |
| *TP*0577 |  | ABC transporter substrate-binding protein*** | insertion (24 nt) | similar to TEN 11q/j |
| *TP*0617a |  | hypothetical protein | insertion (1 nt in C-homolymer region) | gene identical to *TP*CCA_0315 and *TP*LL2_0315 |
| *TP*0651 |  | tetratricopeptide repeat containing protein** | deletion (6 nt) | sequence version similar to *TP*E strains, deletion of this region is present in Cuniculi A  protein elongation of 40 aa at the N-terminus |
| *TP*0733 |  | OprG/OmpW-like ion-channel**** | deletion (6 nt)  MSC (24 nt) | deletion of 2 aa, no similarity to other treponemal sequences |
| *TP*0778 | ***mazG*** | nucleoside-triphosphate pyrophosphatase | mutation in stop codon (substitution, 1 nt) | protein elongation of 11 aa at the C-terminus |
| *TP*0801 |  | A*TP*-dependent Clp protease, A*TP*-binding subunit ClpA | frameshift (deletion, 1 nt) | protein elongation of 37 aa at the C-terminus |
| *TP*0896 |  | hypothetical protein | deletion (17 nt) | pseudogenes in both V3603-13 and *TP*eC  sequence version similar to *TP*E strains, deletion of this region is present in Cuniculi A |
| *TP*0897 | ***tprK*** | *TP*r protein K | insertion (12 nt)  deletion (6 nt)  MSC (3 nt, 45 nt, 41 nt, 136 nt) | insertion of 4 aa  deletion of 2 aa  similar to *TP*A SS14 sequences |
| *TP*0898 | ***recB*** | exodeoxyribonuclease V beta subunit | insertion (3 nt)  MSC (16 nt) | insertion of 1 aa  MSC around the insertion site, no similarity |
| *TP*0966 |  | outer membrane protein** | deletion (6 nt)  MSC (15 nt) | deletion of 2 aa  region similar to *TP*E Fribourg-Blanc |
| *TP*1031 | ***tprL*** | *tpr* protein L | MSC (21 nt) | no similarity |
| *TP*1035a |  | hypothetical protein | frameshift (deletion, 1 nt) | not present in *TP*eC |

*MSC, major sequence changes. MSCs were defined as regions containing heterologous DNA sequence in the length of 15 and more nucleotides; **according to [1]; ***according to BLAST result; ****according to [2]

**References**

1. Naqvi AA, Shahbaaz M, Ahmad F, Hassan MI. Identification of functional candidates amongst hypothetical proteins of *Treponema* *pallidum* ssp. *pallidum*. PLoS One. 2015 Apr 20;10(4):e0124177. doi: 10.1371/journal.pone.0124177. Erratum in: PLoS One. 2018 May 14;13(5):e0197452. doi: 10.1371/journal.pone.0197452. PMID: 25894582; PMCID: PMC4403809.
2. Radolf JD, Kumar S. The *Treponema* *pallidum* Outer Membrane. Curr Top Microbiol Immunol. 2018;415:1-38. doi: 10.1007/82_2017_44. PMID: 28849315; PMCID: PMC5924592.
